# Retarding Oxidative and Enzymatic Degradation of Phenolic Compounds Using Large-Ring Cycloamylose

**DOI:** 10.3390/foods10071457

**Published:** 2021-06-23

**Authors:** Shin-Joung Rho, Saehun Mun, Jiwoon Park, Yong-Ro Kim

**Affiliations:** 1 Center for Food and Bioconvergence, Seoul National University, Seoul 08826, Korea; baboshin@snu.ac.kr (S.-J.R.); cocomi1107@snu.ac.kr (J.P.); 2 Department of Food and Nutrition, Soonchunhyang University, Asan 31538, Korea; saehun73@sch.ac.kr; 3 Department of Biosystems Engineering, Research Institute of Agriculture and Life Sciences, Global Smart Farm Convergence Major, Seoul National University, Seoul 08826, Korea

**Keywords:** cycloamylose, cyclodextrin, phenolic compounds, antioxidant activity, anti-browning effect

## Abstract

The phenolic compounds (PCs) abundant in fruits and vegetables are easily browned by oxygen and browning enzymes, with subsequent destruction of nutrients during food processing and storage. Therefore, natural anti-browning additives are required to control these reactions. The aim of the present study was to investigate the feasibility of cycloamylose (CA) complexation as a way to improve stability of PCs against oxidation and browning enzymes. The complex was prepared by reacting enzymatically produced CA with a degree of polymerization of 23–45 with PCs in aqueous solution. No significant differences were observed between the PCs and their CA complexes in 2,2-Diphenyl-1-picrylhydrazyl (DPPH) radical scavenging experiments. However, the reduction rate of their antioxidant activity was clearly reduced in the presence of CA for as long as 4 weeks. At the studied concentrations, the activity of polyphenol oxidase on all of the tested PC species was inhibited in the presence of CA, although this effect was less evident as the substrate concentration increased. The higher the CA concentration added to apple juice, the lower the variation in the total color difference (ΔE*) during storage, confirming that CA could be used as an effective natural anti-browning agent. Our study is the first to study the potential of CA as a natural material for browning control. The results obtained will provide useful information for active food applications requiring oxidative stability in fruit products.

## 1. Introduction

Phenolic compounds (PCs) are naturally present in almost all plant materials, contributing to their sensorial properties, such as color, taste, aroma, and texture. In addition, PCs have a wide range of physiological activities such as anti-cancer, anti-inflammatory, antibacterial, and antioxidant effects, and thus are widely used as a functional additive in various industries [1,2,3]. However, PCs can act as polyphenol oxidase substrates (PPOS) that can cause browning by oxygen and enzymes during food processing and storage [4]. PCs are structurally unstable due to aromatic rings bearing one or more hydroxyl groups, together with a number of other substituents, being thus easily modified by oxidative enzyme action. In the presence of oxygen, PCs interact with polyphenol oxidase (PPO, EC 1.14.18.1), the main cause of browning, to hydroxylate monophenols (colorless) to diphenols (colorless) [5,6]. Subsequently, quinones (colored), melanin pigments associated with browning, are formed, which reduce the sensory quality and alter the nutritional properties of foods, thereby shortening their shelf-life [5,7,8]. Browning of fruits and vegetables is one of the main causes of quality loss during post-harvest handling and processing [9]. Therefore, controlling them is essential to maintaining the quality and safety of fresh food containing PCs and browning enzymes [10,11,12,13,14].

Several studies have been conducted using cyclodextrin (CD) as an anti-browning agent to control the oxidative and enzymatic degradation of PCs [15,16,17,18,19,20]. Native CD (α-, β-, and γ-CD) are small cyclic glucans composed of six, seven, or eight glucose units that possess hydrophobic inner cavities [21], delaying enzymatic browning by partially complexing the PPO substrate [22,23]. Among those three CDs, β-CD has been more widely studied and applied in food industry, because the application scope of α-CD is small due to its small molecular cavity size and the production cost of γ-CD is high [24]. However, β-CD has a structurally rigid cavity, resulting in relatively poor solubility in water compared to other glucose polymers (approximately 1.85 g per 100 mL) due to its hydrogen bonding [25]. This can result in precipitation with guest molecules at its high concentrations [25,26].

In this study, we presented cycloamylose (CA) as a novel natural anti-browning agent that can compensate for the shortcomings of CD. CA is a large-ring cyclic glucan, composed of a glucose unit with a degree of polymerization (DP) higher than 17, and is produced by treatment of a starch or linear glucan with α-glucanotransferase [27]. It provides a hydrophilic external surface with a relatively hydrophobic cavity and has emerged as a material capable of improving physicochemical properties of unstable functional molecules through complex formation in a similar way to that of small CD [28,29,30]. Differently from CD, CA has various cavity geometries by providing structurally distorted or bent-flipped motifs. Previous studies reported that the CA24 molecules composed of DP 24 have an average radius of gyration of 27.8 Å and gradually increased with increasing DP (41.0 Å at CA55) [31,32]. The native CD has a rigid structure, as mentioned earlier, and has a smaller radius of gyration (6.0 Å for α-CD, 6.7 Å for β-CD, and 7.3 Å for γ-CD, respectively) than those of CA molecules [33]. With a wide range of molecular size and structural flexibility, CA can be more advantageous than CD because of its diverse cavity geometry and higher solubility in cold water (>100 g per 100 mL) [27,34]. In addition, it is less toxic than the CD and does not increase in viscosity even when used in large quantities [33,35].

CA, which has the advantage of being a potential host material for use in complex system, still lacks information on the molecular interactions and capabilities between functional compounds. In previous studies, we confirmed that CA consisting of 23–45 glucose formed a complex through physicochemical interaction with PCs [36]. Fluorescence spectrometry, isothermal titration calorimetry (ITC), nuclear magnetic resonance (NMR) spectroscopy, X-ray diffractometry (XRD), differential scanning calorimetry (DSC), and scanning electron microscopy (SEM) results revealed that CA could accommodate a greater number of PC molecules due to its higher solubility and larger hydrophobic cavities compared to CD [36]. This study, as a follow-up study, investigated whether the complex exhibited an inhibitory effect on browning against the oxidation or PPO, based on the formation of CA and PC complexes demonstrated in our previous study. In addition, the anti-browning effects of CA were evaluated by adding CA at various concentrations to apple juice, the real food product. All experimental results for CA were compared to those for CD that are widely used commercially. Our study is the first study to the potential of CA as a novel natural material for browning control, which will demonstrate the industrial applicability of CA complexes to improve the color stability and delay oxidation and enzymatic processes in fruit products.

## 2. Materials and Methods

### 2.1. Materials

Chlorogenic acid (CHA), caffeic acid (CFA), 3,4-Dihydroxy-l-phenylalanine (l-DOPA), catechol (CT), 4-methylcatechol (4MC), pyrogallol (PY), amylose (from potato), β-cyclodextrin (CD), DPPH (2,2-Diphenyl-1-picrylhydrazyl), and tyrosinase (PPO, EC 1.14.18.1) were purchased from Sigma Aldrich Co. (St. Louis, MO, USA). Pullulan standard P-82 (5900–788,000 Da) was purchased from Showa Denko (Tokyo, Japan). Apples (*Busa* variety) were obtained at commercial maturity from the local market and maintained at low temperature (under 4 °C) until processed. All other reagents were of analytical-reagent grade.

### 2.2. Analysis of Produced Cycloamylose

Cycloamylose (CA) was prepared by treating amylose with *Thermus aquaticus* 4-α-glucanotransferase as in our previous study [36]. The molecular weight distributions CA product was analyzed by high-performance size exclusion chromatography (HPSEC) equipped with a refractive index detector (Prostar, Varian Inc., Palo Alto, CA, USA) and two SEC columns (Shodex OHpak SB-804 HQ and OHpak SB-802 HQ; 8.0 mm ID × 300 mm each, Show Denko K.K., Tokyo, Japan) at a flow rate of 1.0 mL/min. The molecular weight of CA products was assessed using the pullulan standard, α-CD, and β-CD.

### 2.3. Stability of PCs with CA against Oxidation

#### 2.3.1. DPPH Radical Scavenging Activity

The free radical scavenging activity of phenolic compounds (PCs) was measured by the 2,2-diphenyl-1-picrylhydrazyl (DPPH) method, as described by Blois [37] with a slight modification. The complex samples were prepared at the complex formable concentrations (5 mM PCs and 10 mM CA or CD) as reported in our previous study [36]. An aliquot of each complex sample (0.2 mL) was added to 1.8 mL of the DPPH solution under vigorous mixing. After 30 min of reaction, the decrease in absorbance at 525 nm upon free radical bleaching was measured against a 50% ethanol blank to estimate the radical scavenging properties of each sample. The DPPH radical scavenging activity was calculated by the following equation (Equation (1)):

AU (%) = [(A_0_ − A_s_)/A_0_] × 100 (1)

where AU is the radical scavenging activity, A_s_ is the absorbance measured for the different substrates, and A_0_ is the absorbance measured without substrates (blank sample).

#### 2.3.2. Oxidative Stability of PCs with CA

To compare the oxidative stability of the PCs in the presence of CA and CD after 4 weeks, the relevant degradation rate constants (*k_d_*) and half-life (*t*_1/2_) were calculated using simple first-order kinetics, according to the following equation:

*C*_t_ = *C*_0_ exp(-*k_d_t*) (2)

*t*_1/2_ = ln2/*k_d_* (3)

where *C*_t_ is the DPPH radical scavenging activity of the PCs (%) remaining in solution at time *t* (day), *C*_0_ is the initial DPPH radical scavenging activity of PCs at *t* = 0, and *k_d_* is the degradation rate constant (day^−1^). As ln 2 equals 0.693, the *t*_1/2_ is readily calculated from *k_d_*.

The parameters of the first-order kinetic model (Equation (2)) were estimated by the non-linear regression iterative procedure of Sigma Plot (SigmaPlot 10.0 Windows version, Systate Software, Inc., San Jose, CA, USA).

### 2.4. Stability of PCs with CA against Enzymatic Oxidation

The kinetic assays for activity of polyphenol oxidase (PPO), tyrosinase, were performed using six PC substrates by measuring the increase in absorbance due to the formation of oxidation products. The PCs’ concentration was set in the range of 0.1 to 10 mM depending on the *K*_m_ values of free PCs. After adding 0.4 mL of PPO (1 μg/mL final protein concentration) to 1.8 mL of PC solutions and 1.8 mL of 10 mM CA or CD, these solutions were monitored every 15 s for 5 min by a UV/Vis spectrophotometer (UV-1650 PC, Shimadzu, Japan). The increase in absorbance due to product formation by the PPO was measured at 400 nm for CHA, CT, 4MC, and PY, and at 475 nm for CFA and DOPA, respectively. PPO activity was defined as change in absorbance per second (Δ_abs_ s^−1^).

The initial reaction rate (*V*_0_) was determined at each PC concentration by plotting the absorbance vs. time and calculating the nonlinear regression fit of the *V*_0_ vs. [PCs]_0_ data to the Michaelis–Menten equation (Equation (4)):

*V*_0_ = *V*_max_ [S]/*K*_m_ + [S] (4)

The fitting was performed using a commercial nonlinear least-square (Marquardt’s algorithm) method implemented in the Sigma Plot 10.0 program. To obtain the kinetic constants (*K*_m_ and *V*_max_) for each substrate, the plot was transformed into a linear equation considering the double reciprocal Lineweaver–Burk equation (Equation (5)):

1/*V*_0_ = 1/*V*_max_ + *K*_m_/*V*_max_ [S] (5)

### 2.5. Color Evolution Assessment

The apple juice was extracted with a MX2000 blender (Braun GmbH, Kronberg, Germany), immediately collected, and mixed with 25 mL of DW alone (blank) or different concentrations of CA and CD (ranging from 0 to 15 mM). Extraction was replicated (3 times) while blocking light at room temperature.

The instrumental analysis was performed using a colorimeter (Colormate, Scinco Co., Ltd., Seoul, Korea) equipped with the ColorMaster Plus software by measuring the reflectance with a D65 light source, in large-view area mode. The color measured was expressed by the uniform CIE L*, a*, b* (CIELAB) color space values as per the international standard [18]. All the measurements were made at different times during the first 70 min after the materials were dissolved in apple juice, i.e., just after the beginning of enzymatic browning. All the experiments and measurements were performed in triplicate. The total color difference (ΔE*) was calculated from the lightness (L*), redness (a*), and yellowness (b*) parameters using the Hunter–Scofield equation (Equation (6)):

ΔE* = [(ΔL*)^2^ + (Δa*)^2^ + (Δb*)^2^]^1/2^ (6)

In addition, the velocity of ΔE* evolution (*ν*) was calculated by the Michaelis–Menten equation to compare the variation in ΔE* with increasing CA or CD concentration.

### 2.6. Statistical Analysis

All data were recorded as mean ± standard deviation and analyzed by IBM SPSS statistics for Windows, version 23.0 (IPM Corp., Armonk, NY, USA). A one-way ANOVA test followed by a Tukey’s test was performed to identify statistical significances (*p* < 0.05).

## 3. Results and Discussion

### 3.1. Synthesis of CA

**Figure 1:**
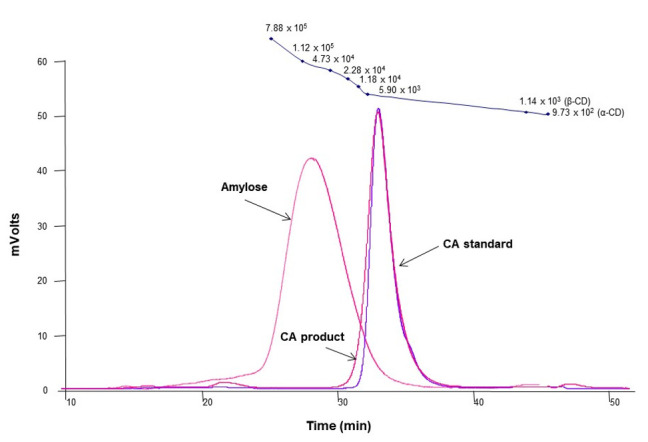
The molecular weight distribution of amylose, cycloamylose (CA) standard, and the CA product. At the top of the graph, the molecular weight values measured with pullulan standard, α- and β-cyclodextrin (CD) were plotted.

The molecular weight distribution of cycloamylose (CA) produced by treating *Thermus aquaticus* 4-α-glucanotransferase (4αGTase) on amylose showed a sharp peak at 30–39 min similar to that of the CA standard (Ezaki Glico Co., Ltd., Osaka, Japan) with the peak shifted to the right compared to amylose (Figure 1). As shown in Supplementary Materials Figure S1, the 4αGTase (EC 2.4.1.25) catalyzes the intramolecular transglycosylation (cyclization) towards linear α-1,4-glucans (amylose), yielding novel cyclicα-1,4-glucans (CA) [27,38,39]. Although cyclodextrins (α-, β-, andγ-CD) can also be produced by the cyclization reaction of cyclodextrin glycosyl transferase (EC 2.4.1.19, CGTase), they are mainly composed of six, seven, and eight-D glucose units that are less than those of CA [40,41,42]. On the HPSEC chromatogram, the CA elution peak appeared at 33.16 min, corresponding to an average molecular weight of approximately 5500 Da [36] and showed a higher molecular weight distribution than native CD (972–1297 Da) [43]. Previously, we confirmed that the CA product used in this study had a DP distribution ranging from 23 to 45 by MALDI-TOF MS [36]. A previous study revealed that most of the CA molecules exist in two short helical structures connected by two elongated loops of different sizes [32]. Therefore, it could be inferred that CA produced in this study was mostly larger than CD and formed one or more cavities of different sizes and shapes through folding and bend-flips.

### 3.2. Effect of CA on the Oxidative Stability of PCs during Storage

**Figure 2:**
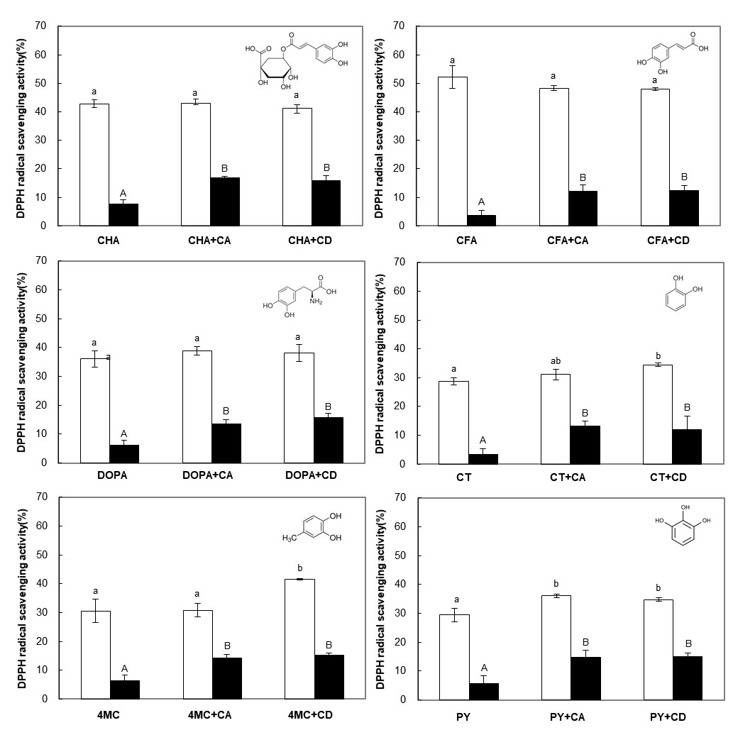
DPPH radical scavenging activities of phenolic compounds (CHA, chlorogenic acid; CFA, caffeic acid; DOPA, 3,4-Dihydroxy-l-phenylalanine; CT, catechol; 4MC, 4-methylcatechol; PY, pyrogallol) detected at 0 day (empty bar) and after 4 weeks (black bar) in the absence and presence of cyclic glucans (cycloamylose (CA) and cyclodextrin (CD)). The molecular structure of each phenolic compound is shown in the upper right of the bar graph. The error bars represent the mean and standard deviations (*n* = 3). Bars with the different letter (a and b for white bars; A and B for black bars) are significantly different based on Tukey’s test at *p* < 0.05.

To investigate the effect of CA on the oxidative stability of PCs during storage, the DPPH radical scavenging activity (antioxidant activity) was measured in the presence of CA. The results were expressed as the antioxidant activity after 4 weeks, which was also compared with that of the CD-PC complexes and a control (Figure 2 and Supplementary Materials Figure S2). In this study, all experiments were conducted in 50% ethanol conditions, because CA precipitated at ethanol percentages above 50% and the DPPH reagent precipitated at ethanol concentrations below 50% [44].

The DPPH radical scavenging activity (%) of PCs before storage (Figure 2, empty bar) showed a similar tendency to previous reports on the antioxidant profiles of PCs in the order of CFA > CHA > DOPA ≈ 4MC > PY ≈ CT [45,46]. The PCs used in this study were structurally different in three ways: (a) Numbers and positions of the hydroxyl groups, (b) the presence of acrylic functional groups (CHA and CFA), and (c) the presence of a quinic acid moiety (CHA). In general, the antioxidant activity of these PCs depends on several factors, including the number and arrangement of hydroxyl groups, the degree of conjugation between them, and the nature of the radicals in the ring structure [44,47]. Our results showed that the antioxidant activity of PCs was relatively high depending on the type and presence of functional groups attached to the aromatic ring along with a dihydroxyl group (Figure 2, empty bar). In the presence of CA, the scavenging ability of the tested PCs toward DPPH before storage (at 0 day) did not change, almost significantly, in comparison with that of the control. Several studies have reported that the inclusion process with CD has little influence on the antioxidant activity of some PCs [48,49]. In addition, it was reported that DPPH reached reaction equilibrium more slowly with the complex form than with free form [50]. Therefore, the active groups in the PC molecules may not be interfered with by the CA cavity when reacting with DPPH radicals, but they may not be effective due to not reaching a reaction equilibrium.

During storage for 4 weeks at room temperature, the antioxidant activity of the PCs in both free and complexed forms decreased gradually (Supplementary Materials Figure S2). As mentioned above, the hydroxyl groups of PCs, a main factor causing the antioxidant activity, was easily oxidized during storage, resulting in a decrease in antioxidant activity [46,51]. After 4 weeks of storage, the antioxidant activity of PCs was reduced by more than 80% for the control, while samples with cyclic glucans decreased relatively more slowly (Figure 2, black bar). As a result of comparing the degradation rate constant (*k_d_*) of the antioxidant activity calculated using Supplementary Materials Figure S2, when a PC interacted with cyclic glucans, degradation of the PC molecules occurred more slowly during storage compared with the control sample (Table 1). The half-lives of the complex form were also about 2.5 times longer on average compared to those of control sample, delaying the decrease in antioxidant activity. This means that both CD and CA were able to improve the oxidative stability of active groups that are readily oxidized in PC molecules by complexation, as reported previously [36,49]. In particular, the *k_d_* values of CT (about 4.3 times) and 4 MC (about 3.1 times) clearly decreased in the presence of CA during storage, possibly due to the structural changes in CA when interacting with them. The macrocycle of CA is highly distorted or bend-flipped in the free state, but its structure could be unraveled or become flexible when the complex is formed in an aqueous solution [27,32]. In the antioxidant experiments, it was conclusively shown that the interaction with cyclic glucans stabilized the PCs to retard the decrease of their antioxidant capability during storage.

### 3.3. Effect of CA on PCs Stability against Enzymatic Oxidation

**Figure 3:**
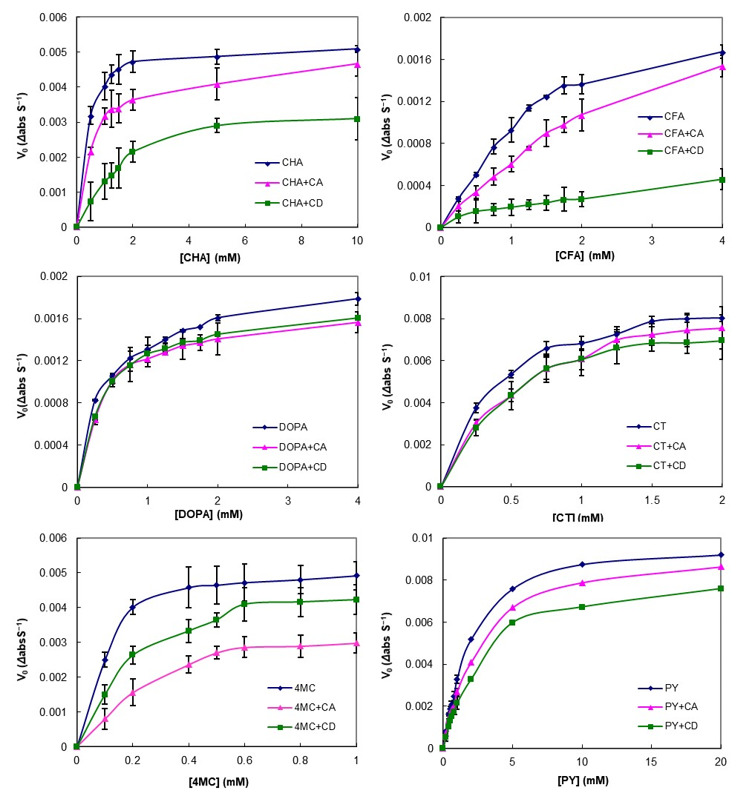
Effect of cyclic glucans (cycloamylose (CA) and cyclodextrin (CD)) on phenolic compounds (CHA, chlorogenic acid; CFA, caffeic acid; DOPA, 3,4-Dihydroxy-l-phenylalanine; CT, catechol; 4MC, 4-methylcatechol; PY, pyrogallol) oxidation catalyzed by polyphenol oxidase (PPO). Symbols represent experimental data (± SEM). Lines were the Michaelis–Menten curves fitted to the experimental data. The error bars indicate standard deviation (*n* = 3) and statistically significant differences based on Tukey’s test (*p* < 0.05).

The effect of CA on the enzymatic oxidation of PCs was measured using tyrosinase as the polyphenol oxidase (PPO) and the results are displayed in Figure 3. In the control group (free PC), a steep increase was observed even at low PC concentrations, meaning that browning caused by the enzyme progressed rapidly. However, the reaction rates for all PC concentrations studied in the presence of cyclic glucan were lower compared with the control and were strongly substrate dependent. For CT and DOPA, the reaction rate decreased by approximately the same amount for both CA and CD, whereas, in the cases of CHA, CFA, and PY, it was more strongly inhibited by CD than by CA. Interestingly, the reaction rate of 4MC was significantly lowered in the presence of CA. In our previous study, the binding constants (*K_a_*) of 4MC and CA were higher than PY and CT, but not higher than CHA and CFA [36]. This result was a different trend from previous studies that suggested that the extents of the enzyme reaction rate correlated with the affinity of the substrates participating in the complex formation [22,52]. Browning inhibitory properties via complex formation were typically due to PPO substrate sequestration or depletion [16,22,52,53]. However, some studies have argued for PPO inactivation by interaction with the hydrophobic amino acid side chain of PPO and the host molecules [20,54]. Our results might be attributed to the inhibitory mechanism of direct PPO inactivation by CA.

In order to characterize the action of the enzyme on PCs and to confirm the inhibition of PCs enzymatic oxidation by CA, the apparent kinetic constants (*K*_m_ and *V*_max_) of PPO for the different PCs were calculated by nonlinear fitting to Equation (4) and linear fitting to Equation (5). The values obtained for the six PCs tested are summarized in Table 2. Generally, both the linear and nonlinear fitting results showed mixed inhibition patterns for CD and CA, where *V*_max_ decreased and *K*_m_ increased, even though DOPA and CT showed less change. The inhibition patterns for both CA and CD were similar to the mixed inhibition pattern for all substrates (Table 2). These results showed that CA can be considered as a good inhibitor of PPO, having a similar efficiency to CD. CA could inhibit the enzyme activity by reducing the amount of free PC molecules by complexation, thereby preventing their oxidation to quinone species and subsequent polymerization to brown pigments. Previous studies reported that the inhibition of PPO activity by complex formation of CD with CHA was attributed to reduce the concentration of free substrates as a result of a complexation-dependent inhibition mechanism with a mixed-type pattern [20,54]. Furthermore, they reported two possible mechanisms for the inhibitory effect of CD on the PPO activity: Via reduction of the free substrate and via reduction of the catalytic activity by direct interaction of the enzyme with CD. Although further studies using various CA concentrations are required for a clear explanation of the inhibitory mechanisms, the inhibitory effect of CA on PPO activity was considered to occur via a mechanism similar to that of CD because CA can also form intermolecular interaction with the PC moieties.

In conclusion, in the presence of CA and CD, the reaction rate of PCs with PPO was reduced, indicating the inhibition of enzymatic oxidation and, consequently, a reduction of the quinone products. Although the mechanisms for enzymatic browning prevention by CA are not well understood yet, the inhibition seems to occur by direct and/or indirect interference with PPO catalytic activity for the oxidation of PCs. These results point to the feasibility of utilizing CA in browning-prone foods to improve the stability and functionality of various functional compounds—if the optimum conditions and reagents can be determined.

### 3.4. Effect of CA on the Color Stability of Apple Juice

**Figure 4:**
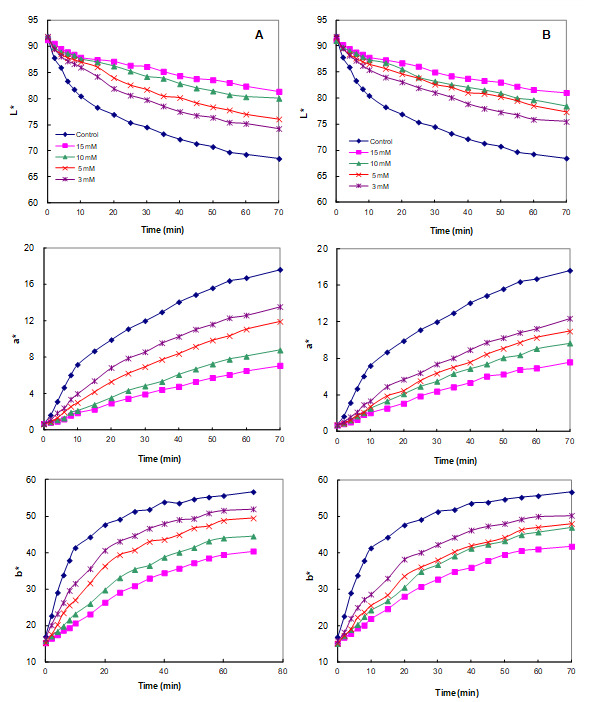
The variation of lightness (L*), redness (a*), and yellowness (b*) value of apple juice in the absence (control) and presence (concentrations of 3, 5, 10, and 15 mM) of (**A**) cyclodextrin (CD) or (**B**) cycloamylose (CA). Each data point is the mean of three replicates.

PPO inhibition experiments of the interaction of the CA identified in the above section with PC may vary significantly from the conditions observed in real food and may reduce or cancel the effect as these studies are performed under controlled conditions (temperature, concentration, or pH) [55]. Therefore, the inhibitory effect of CA was confirmed by measuring whether the color stability of the juice was actually improved by adding CA directly to the juice containing large amounts of PPO and PCs. As can be seen in Figure 4, the lightness (L*) values of the control apple juice decayed significantly and rapidly over 70 min due to browning of the juice. However, the addition of CA significantly retarded changes in the L* value, indicating that CA was effective in delaying the browning of apple juice. With respect to the other tested parameters, Figure 4A showed the evolution of a* and b* in the absence and presence of CA (at concentrations of 0, 3, 5, 10, and 15 mM). In the absence of CA, the a* value substantially increased, turning the apple juice redder with the increasing reaction time, while increasing concentrations of CA led to significantly lower values of a*. The value of b* rapidly increased toward the yellow range at all the tested concentrations of CA, in contrast to previously published results for apple juice [17]. This different trend was due to different apple cultivars and maturity. As shown in Figure 4B, the trends of the scalar (L*, a*, and b*) values after the addition of CD were very similar to those obtained with CA. Increasing concentrations of CD (ranging from 0 to 15 mM) led to significantly lower values of a* and higher values of L* and b* relative to the control.

**Figure 5:**
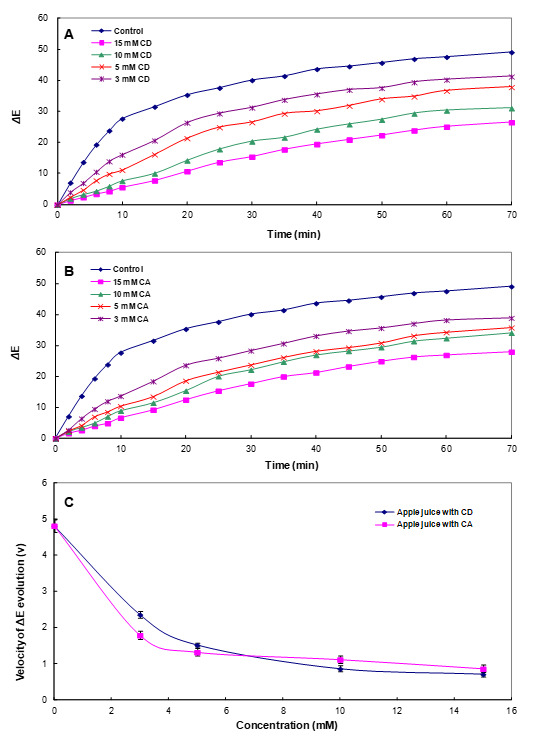
The evolution of total color difference (ΔE*) in apple juice in the absence and presence of (**A**) cyclodextrin (CD) and (**B**) cycloamylose (CA). (**C**) The velocity of ΔE* evolution (*ν*). Each data point is the mean of three replicates.

In the food industry, the colorimetric parameter ΔE* is widely used to characterize the variations in color during food storage and processing [19,56]. Therefore, to confirm the slowing-down of enzymatic browning induced by CA, the total color difference (ΔE*) for the apple juice was also calculated in the presence of increasing concentrations of CA during storage, as shown in Figure 5. In the control, ΔE* rapidly increased in the first 10 min; after this time, the increase was significantly slower up to 70 min. The degradation of the initial color observed in the control was mostly due to the sharp drop in both L* and b* magnitudes. As can be observed in Figure 5B, in the presence of CA, tΔhe E* of the apple juice color exhibited a smaller variation after 70 min with a strong dependence on the CA concentration. The effect of CD on ΔE* was also very similar to that described for CA (Figure 5A). In addition, comparing the velocity of the ΔE* evolution (*ν*), which represents the variation rate in ΔE* with increasing CA and CD concentrations, CA significantly delayed the browning rate for apple juice compared with CD, especially at low concentrations (Figure 5C and Supplementary Materials Figure S3). These results demonstrated that CA addition considerably delayed the enzymatic browning of apple juice, indicating that CA prevented the PCs naturally present in apple juice from reacting with oxygen and enzymes (PPO) by the interaction.

Until recently, studies on anti-browning agents via complex formation have mostly focused on CD [11]. Since CA is a cyclic glucan with various DP other than small CD, the cavity geometry is different, and it has the advantage of high solubility in cold water [57]. Therefore, CA could more efficiently protect the browning substrates, which has different sizes and structures contained in juice. Furthermore, CA can be considered as nutritional as starch does not show toxicity and can be used without changing the viscosity at a high concentration [33,35]. Since the *ν* value during storage was strongly dependent on the CA concentration, CA, even at a high concentration, may be usable as a stable, natural browning agent.

## 4. Conclusions

In the presence of CA, the antioxidant capacity of PCs was maintained for longer during a storage period of 4 weeks, and the action of tyrosinase (a PPO enzyme) on the substrates was reduced. Remarkably, CA addition improved the color stability of apple juice. The CA-PC complexes generally showed no significant differences compared to CD-PC complexes, indicating the suitability of CA for industrial use as a new natural anti-browning agent that may overcome the disadvantages of CD. Unlike CA, which has high solubility (>100 mM), CD precipitates at concentrations higher than those used in this study, thus resulting in lower inhibitory effects. Considering these results, we propose that CA has great potential as a new stabilizer for PCs and a natural anti-browning agent for apple juice, since it provides a bioactive ingredient with health-promoting properties while reducing the enzyme browning activity. This study worked to investigate the effect of cyclic glucans on stabilizing PC in fruit juice for a wide range of concentrations and is the first study to demonstrate the feasibility of CA as an anti-browning agent. For the industrial application of CA, it will be necessary to determine the optimum concentration of CA with the consideration of economical, dietetical, and sensory aspects, which will be an important next step.

## Figures and Tables

**Table 1:** Estimated degradation rate constants *(k_d_*) and half-life (*t*_1/2_) of DPPH radical scavenging activity for PCs in the absence and presence of CA and CD.

Host Molecules ^1^	*k_d_* (Day^−1^)					
	CHA ^2^	CFA	DOPA	CT	4MC	PY
Control	0.075 ± 0.0081 ^b^	0.161 ± 0.0024 ^b^	0.089 ± 0.0026 ^b^	0.102 ± 0.0020 ^c^	0.079 ± 0.0018 ^c^	0.061 ± 0.0016 ^b^
CA	0.037 ± 0.0015 ^a^	0.046 ± 0.0025 ^a^	0.036 ± 0.0015 ^a^	0.024 ± 0.0016 ^a^	0.026 ± 0.0013 ^a^	0.030 ± 0.0015 ^a^
CD	0.036 ± 0.0017 ^a^	0.054 ± 0.0026 ^a^	0.029 ± 0.0017 ^a^	0.040 ± 0.0017 ^b^	0.043 ± 0.0017 ^b^	0.037 ± 0.0018 ^a^
**Host** **Molecules**	***t*_1/2_ (Day)**					
	**CHA**	**CFA**	**DOPA**	**CT**	**4MC**	**PY**
Control	9.34 ± 1.424 ^a^	4.30 ± 0.091 ^a^	7.79 ± 0.322 ^a^	6.80 ± 0.185 ^a^	8.81 ± 0.29 ^a^	11.39 ± 0.430 ^a^
CA	18.97 ± 1.099 ^b^	15.04 ± 1.151 ^b^	19.50 ± 1.146 ^b^	29.24 ± 2.728 ^b^	27.14 ± 1.979 ^b^	23.31 ± 1.649 ^b^
CD	19.57 ± 1.325 ^b^	12.79 ± 0.866 ^b^	23.65 ± 1.934 ^b^	17.58 ± 1.038 ^c^	16.14 ± 0.908 ^c^	18.88 ± 1.284 ^b^

^1^ Host molecules: CA = cycloamylose, CD = cyclodextrin.^2^ Guest molecules: CHA = chlorogenic acid, CFA = caffeic acid, DOPA = 3,4-Dihydroxy-l-phenylalanine, CT = catechol, 4MC = 4-methylcatechol, PY = pyrogallol. Values were expressed as mean ± SD (*n* = 3). The results marked with the different letter in a column are significantly different based on Tukey’s test at *p* < 0.05.

**Table 2:** Apparent kinetic parameters of tyrosinase toward PCs in the absence and presence of cyclic glucans.

Guest Molecules ^2^	V_max_^3^						K_m_					
	Non-Linear Fitting ^4^			Linear Fitting ^5^			Non-Linear Fitting			Linear Fitting		
	C ^1^	CA	CD	C	CA	CD	C	CA	CD	C	CA	CD
CHA	5.3	4.8	3.8	5.4	4.8	4.4	0.30	0.56	1.81	0.33	0.59	2.45
CFA	2.5	2.4	0.7	3.3	2.3	0.5	1.56	2.75	2.90	2.67	2.12	1.68
DOPA	1.9	1.7	1.7	1.8	1.7	1.8	0.36	0.38	0.38	0.30	0.41	0.40
CT	9.8	9.7	9.0	9.7	9.8	9.5	0.40	0.56	0.50	0.40	0.57	0.59
4MC	5.5	4.1	5.3	5.7	5.3	5.7	0.10	0.32	0.22	0.12	0.55	0.27
PY	10.5	10	8.9	10.8	9.1	9.3	2.22	2.74	3.02	2.41	2.41	3.28

^1^ Host molecules: C = control, CA = cycloamylose, CD = cyclodextrin.^2^ Guest molecules: CHA = chlorogenic acid, CFA = caffeic acid, DOPA = 3,4-Dihydroxy-L-phenylalanine, CT = catechol, 4MC = 4-methylcatechol, PY = pyrogallol. ^3^ V_max_ and K_m_ values were expressed as Δ_abs_ S^−1^/10 and mM, respectively. ^4^ Values obtained by non-linear fitting to Equation (4). ^5^ Values obtained by linear fitting to Equation (5).

## Data Availability

The data presented in this study are available on request from the corresponding author.

## Supplementary Materials

The following are available online at https://www.mdpi.com/article/10.3390/foods10071457/s1, Figure S1: The catalytic actions of 4-α-glucanotransferase (4αGTase) on linear α-1,4 glucans (amylose), Figure S2: The variation in the DPPH radical scavenging activity of PCs in the absence and presence of CA and CD for 4 weeks. Each data point is the mean of three replicates, Figure S3: Color change according to the concentration of cyclic glucan (CD (left) and CA (right)) added in apple juice after (A) 20 min, (B) 40 min, and (C) 60 min.
