# Supplementary material for: Retarding Oxidative and Enzymatic Degradation of Phenolic Compounds Using Large-Ring Cycloamylose
Source: Foods. 2021 Jun 23;10(7):1457. doi: 10.3390/foods10071457 (PMC8303965; doi:10.3390/foods10071457)
Supplement: Supplementary file 1 [file foods-10-01457-s001.zip › foods-1263995-supplementary.pdf]

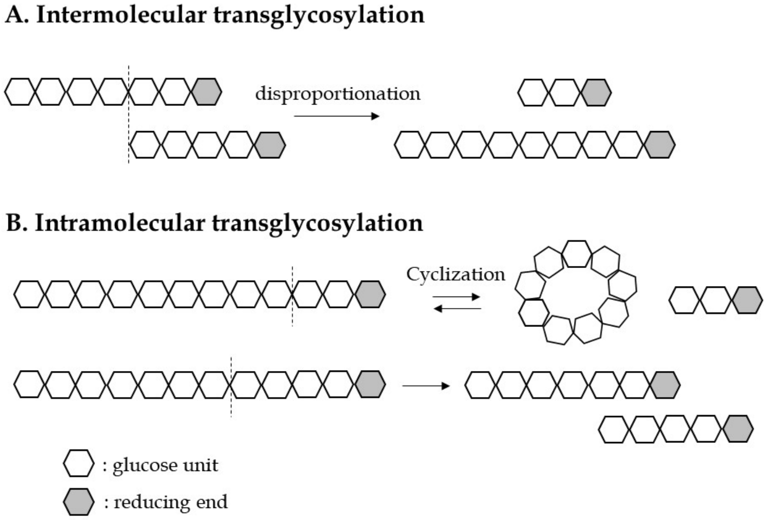

Figure S1. The catalytic actions of 4-α-glucanotransferase (4αGTase) on linear α-1,4 glucans (amylose).

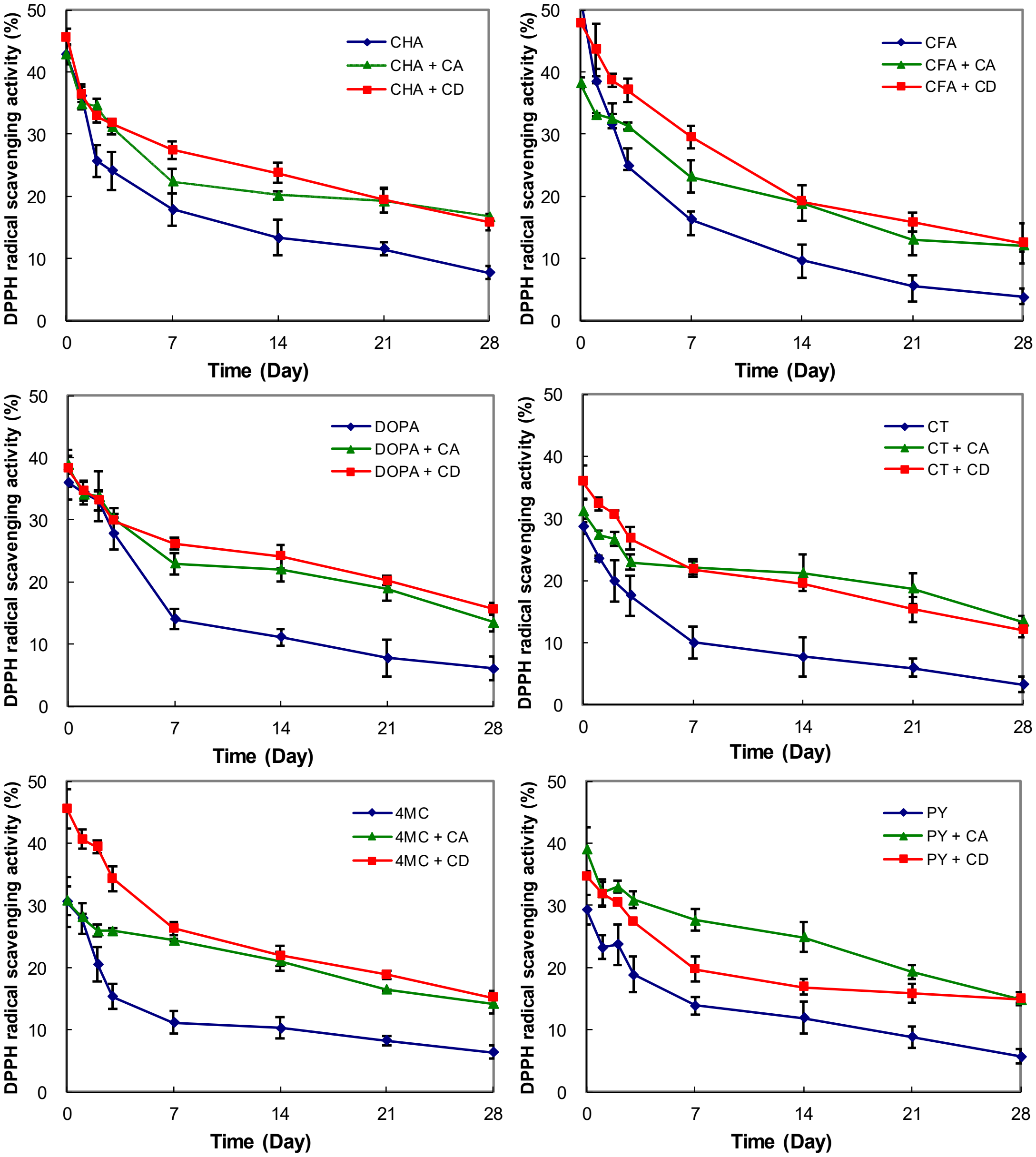

Figure S2. The variation in the DPPH radical scavenging activity of PCs in the absence and presence of CA and CD for 4 weeks. Each data point is the mean of three replicates.

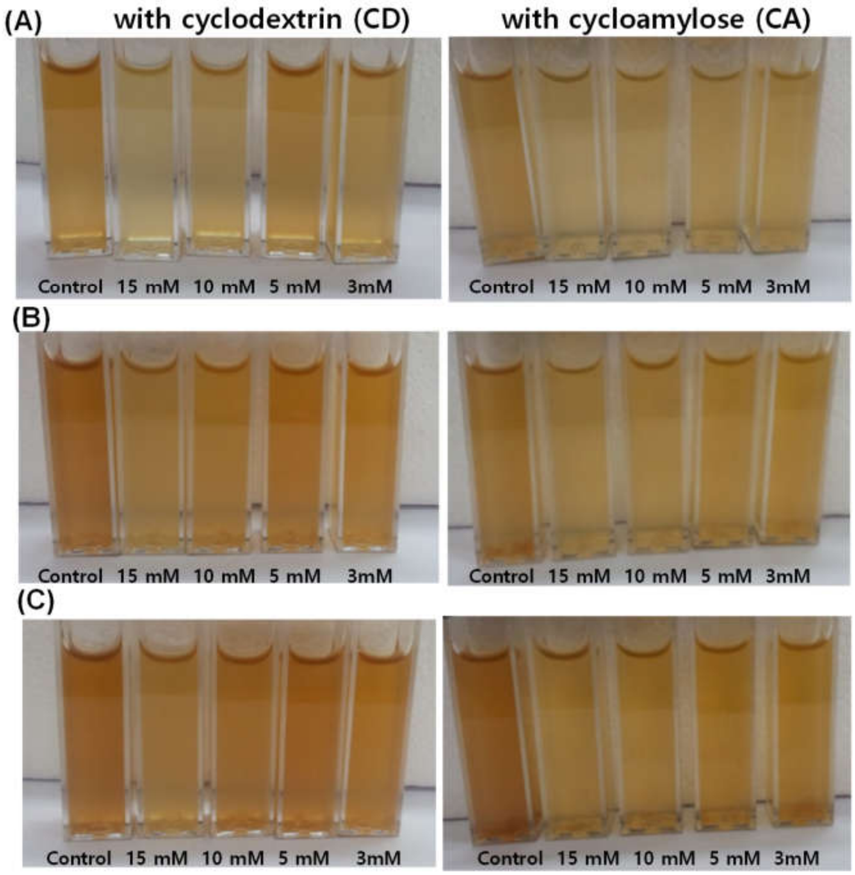

Figure S3. Color change according to the concentration of cyclic glucan (CD (left) and CA (right)) added in apple juice after (**A**) 20 min, (**B**) 40 min, and (**C**) 60 min.
